# Exogenous Wnt1 Prevents Acute Kidney Injury and Its Subsequent Progression to Chronic Kidney Disease

**DOI:** 10.3389/fphys.2021.745816

**Published:** 2021-11-08

**Authors:** Xue Hong, Yanni Zhou, Dedong Wang, Fuping Lyu, Tianjun Guan, Youhua Liu, Liangxiang Xiao

**Affiliations:** ^1^State Key Laboratory of Organ Failure Research, National Clinical Research Center of Kidney Disease, Division of Nephrology, Nanfang Hospital, Southern Medical University, Guangzhou, China; ^2^Department of Nephrology, Xiamen Hospital Affiliated to Beijing University of Chinese Medicine, Xiamen, China; ^3^Department of Nephrology, Zhongshan Hospital Affiliated to Xiamen University, Xiamen, China; ^4^Department of Endocrinology and Diabetes, The First Affiliated Hospital of Xiamen University, Xiamen, China; ^5^Department of Pathology, University of Pittsburgh School of Medicine, Pittsburgh, PA, United States

**Keywords:** acute kidney injury, chronic kidney disease, Wnt1, β-catenin, ischemia-reperfusion injury

## Abstract

Studies suggest that Wnt/β-catenin agonists are beneficial in the treatment of acute kidney injury (AKI); however, it remains elusive about its role in the prevention of AKI and its progression to chronic kidney disease (CKD). In this study, renal Wnt/β-catenin signaling was either activated by overexpression of exogenous Wnt1 or inhibited by administration with ICG-001, a small molecule inhibitor of β-catenin signaling, before mice were subjected to ischemia/reperfusion injury (IRI) to induce AKI and subsequent CKD. Our results showed that *in vivo* expression of exogenous Wnt1 before IR protected mice against AKI, and impeded the progression of AKI to CKD in mice, as evidenced by both blood biochemical and kidney histological analyses. In contrast, pre-treatment of ICG-001 before IR had no effect on renal Wnt/β-catenin signaling or the progression of AKI to CKD. Mechanistically, *in vivo* expression of exogenous Wnt1 before IR suppressed the expression of proapoptotic proteins in AKI mice, and reduced inflammatory responses in both AKI and CKD mice. Additionally, exogenous Wnt1 inhibited apoptosis of tubular cells induced by hypoxia-reoxygenation (H/R) treatment *in vitro*. To conclude, the present study provides evidences to support the preventive effect of Wnt/β-catenin activation on IR-related AKI and its subsequent progression to CKD.

## Introduction

Acute kidney injury (AKI) is a clinical condition due to a rapid loss of renal function by various injuries, such as drugs, ischemia, sepsis, and toxins (Chawla and Kimmel, 2012; Scholz et al., 2021). AKI is associated with high incidence of morbidity and mortality, responsible for approximately 2 million deaths each year worldwide (Belayev and Palevsky, 2014). Particularly, AKI is a well-recognized independent predicator for the development of chronic kidney disease (CKD) or end-stage renal disease (ESRD), which have placed a huge economic burden on the health care system (Leung et al., 2013; Belayev and Palevsky, 2014; Kurzhagen et al., 2020). Unfortunately, there are no effective drugs for the prevention and treatment of AKI.

Wnt/β-catenin signaling plays fundamental roles in organogenesis, tissue homeostasis, and development of various diseases, including kidney diseases (Soni, 2021). β-Catenin has dual roles by serving as both a structural protein and a transcription regulator (MacDonald et al., 2009). The membrane-bound β-catenin is part of the adherens junction complex and contributes to cell-cell interaction, whereas the cytosolic β-catenin is phosphorylated by glycogen synthase kinase-3β (GSK-3β) and targeted for ubiquitination and proteasomal degradation. However, such degradation of β-catenin could be rescued by the Wnt ligands, a family of secreted glycoproteins with diverse biological activities. The binding of Wnt stabilizes β-catenin, which translocates into the nucleus and acts as a transcription factor for gene regulation. Notably, Wnt/β-catenin signaling is relatively silenced in uninjured adult kidney, but is re-activated during acute and chronic renal injury (Huffstater et al., 2020). Activation of Wnt/β-catenin at the early stage of AKI reduces kidney injury and favors the recovery of renal function, whereas sustained Wnt/β-catenin activation could drive the progression from AKI to CKD (Xiao et al., 2016; Huffstater et al., 2020). Therefore, Wnt/β-catenin signaling is a double-edged sword in kidney injury, and warrants a better understanding. Notably, it remains elusive about the role of Wnt/β-catenin activation in the prevention of AKI and its subsequent AKI-CKD progression.

Renal ischemia-reperfusion injury (IRI) is considered the major cause of AKI. Therefore, renal IRI animal model has been commonly used for the research of AKI and AKI-CKD progression (Bonventre and Yang, 2011). Both pharmacologic and genetic approaches have been used to elucidate the role of Wnt/β-catenin activation in IRI models of AKI and CKD, respectively. Lithium and GSK-3β inhibitors are commonly used to pharmacologically activate Wnt/β-catenin signaling in preclinical AKI models, but it should be noted about their β-catenin-independent effects (Bao et al., 2014). Genetic modifications of GSK-3β, β-catenin, or Wnt ligands (Wnt1 and Wnt9a) have been used to elucidate the role of Wnt/β-catenin activation in AKI and CKD (Bonventre and Yang, 2011; Bao et al., 2014; Zhou et al., 2013, 2018; Liu et al., 2020). Nonetheless, studies on the role of Wnt/β-catenin signaling in the prevention of AKI are quite limited. The present study aimed to elucidate the role of Wnt/β-catenin signaling in the prevention of AKI and its subsequent progression to CKD.

## Materials and Methods

### Animal Study

Male C57BL/6 mice weighing approximately 22–25 g were purchased from Vital River Laboratory Animal Technology (Beijing, China), and maintained in the animal facility (temperature: 22 ± 2°C, humidity: 60 ± 5%, 12h dark/light cycle) at Southern Medical University (Guangzhou, China) with water and food *ad libitum*. Animal studies were approved by the Animal Ethical and Welfare Committee at Southern Medical University. All animals were treated in accordance with the Guide for the Care and Use of Laboratory Animals.

The induction of AKI and CKD in mice was described in our previous studies (Xiao et al., 2016; Zhou et al., 2018). Bilateral renal ischemia/reperfusion injury (BIRI) has been widely used to induce AKI in mice, whereas it increases mortality during the progression of AKI to CKD. Therefore, unilateral renal ischemia/reperfusion injury (UIRI) is used for the research on the progression of AKI to CKD. Briefly, a midline abdominal incision was made in mouse under general anesthesia, and bilateral or left renal pedicles were clipped for 30 min using microaneurysm clamps (item no. 18051-35; Fine Science Tools, Cambridge, United Kingdom). During the surgery, body temperature was maintained approximately 37–38°C using a temperature-controlled heating system.

To evaluate the preventive effect of Wnt/β-catenin activation on AKI, mice were injected with 1 mg/kg hemagglutinin (HA)-tagged Wnt1 expression vector (pHA-Wnt1, Upstate Biotechnology) or empty vector (pcDNA3) 2 days before BIRI using the hydrodynamic-based gene transfer technique as reported previously (Xiao et al., 2016). Blood and kidney tissues were collected 1 day after renal BIRI.

To evaluate the effect of early Wnt/β-catenin activation on the progression of AKI to CKD, mice were either subjected to daily hydrodynamic tail vein injection of Wnt1 expression plasmids at 1 mg/kg or daily intraperitoneal injection of ICG-001 (847591-62-2, Chembest, Shanghai, China) at 5 mg/kg for 3 days before UIRI. Blood and kidney tissues were collected 11 days after UIRI. In our preliminary study, 2 days of Wnt1 injection could induce the expression of Wnt1 in AKI mice (data not shown). It should be noted that 3 days of Wnt1 injection was chosen to ensure and lengthen the expression of Wnt1 during the progression of AKI to CKD.

### Cell Culture and Treatment

Human proximal tubular epithelial cell line (HKC-8) was provided by Dr. L. Racusen at the Johns Hopkins University (Baltimore, MD, United States). Cell culture were performed as previously described (Zhou et al., 2013). HKC-8 cells were treated with human recombinant Wnt1 (SRP4754; Sigma-Aldrich, St. Louis, MO, United States) at 100 ng/mL or ICG-001 (847591-62-2, Chembest, Shanghai, China) at 10 μM for 4 h. Cells were then incubated in a hypoxia workstation (X3-CK, Biospherix) at 1% pO2 for 48 h. Next, cells were re-oxygenated for 2 h before collection for various analyses.

### Apoptosis Assay

Cells were trypsinized, collected, and washed with PBS. PE Annexin V staining was performed according to the manufacturer protocol. A cytometric analysis was performed with a flow cytometer (BD FACSCanto II, San Jose, CA, United States) to measure the apoptosis rates by detecting the relative amount of PE Annexin V positive and 7-AAD negative cells. Each assay was performed in triplicate.

### Creatinine and Blood Urea Nitrogen Assay

Creatinine levels in serum and urine as well as blood urea nitrogen (BUN) levels were measured using an automatic biochemical analyzer (AU480, Beckman-Coulter Inc., Brea, CA, United States).

### Histology and Immunohistochemical Staining

Kidney sections and immunohistochemical staining were performed as previously described (Liu et al., 2020). Primary antibodies included rabbit polyclonal anti–Wnt1 (ab15251; Abcam, Inc.), rabbit polyclonal anti-β-catenin (ab15180; Abcam, Inc.), and rabbit polyclonal anti-fibronectin (F3648; Sigma-Aldrich).

### Western Blot Analysis

Western blot analysis was performed as described previously (Zhou et al., 2019). Primary antibodies included rabbit polyclonal anti-fibronectin (F3648; Sigma-Aldrich), mouse monoclonal anti-β-catenin antibody (610154; BD Transduction Laboratories), mouse monoclonal anti-α-SMA antibody (A2547; Sigma-Aldrich), rabbit polyclonal anti-Wnt1 (ab15251; Abcam), mouse anti-α-tubulin (T9026; Sigma-Aldrich), mouse anti-PAI-1 antibody (AF3828;R&D Systems), anti-active β-catenin (#05–665; EMD Millipore), anti-Fas ligand (FasL) (SC -6237; Santa Cruz, CA, United States), p53 (#2524S; CST), Bax (SC-20067; Santa Cruz), p65 (#8242S; CST), p-p65 (#3033S; CST), PCNA (#2586S; CST).

### RNA Extraction and qPCR Analysis

Total RNA isolation and qPCR were carried out as previously described. The mRNA levels of various genes were normalized with β-actin. The primer sequences of the genes are listed in Supplementary Table 1.

### Statistical Analysis

All data were expressed as mean ± SEM. The data were statistically analyzed using Sigma Stat software (Jandel Scientific Software, San Rafael, CA, United States). Comparison between groups was made using one-way ANOVA, followed by the Student–Newman–Keuls test. *P* < 0.05 was considered significant.

## Results

### *In vivo* Expression of Exogenous Wnt1 Before IR Prevents Acute Kidney Injury and Activates Renal β-Catenin in Mice

**FIGURE 1:**
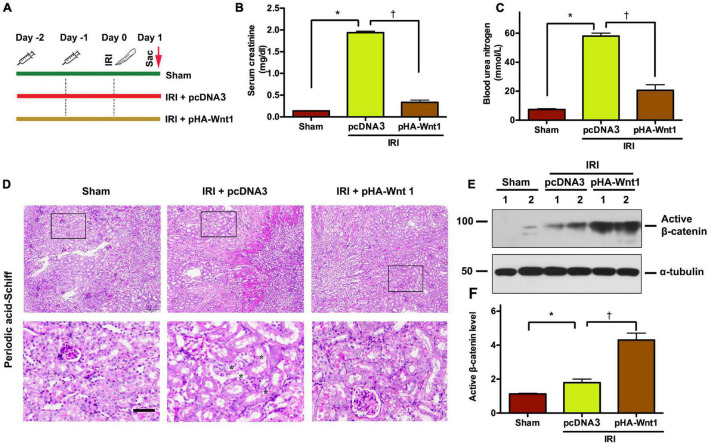
*In vivo* expression of exogenous Wnt1 before IR prevents AKI and activates renal β-catenin in mice. **(A)** Mouse treatment groups. Mice were treated with either pcDNA3 (IRI + pcDNA3) or pHA-Wnt1 (IRI + pHA-Wnt1) plasmid *via* hydrodynamic tail vein injection at 2 days before BIRI. Serum creatinine levels **(B)** and blood urea nitrogen (BUN) levels **(C)** were measured. **(D)** Representative micrographs of kidney morphology at 1 day after BIRI. Kidney sections were subjected to Periodic acid–Schiff staining. Boxed areas are enlarged. Arrows indicate renal tubules. Scale bar, 200 μm. **(E,F)** Western blot analysis of active β-catenin in kidneys of mice. *P < 0.05 versus Sham controls (*n* = 5). ^†^*P* < 0.05 versus IRI injected with pcDNA3 plasmid (*n* = 5).

To determine the role of Wnt/β-catenin activation in AKI prevention, mice were administered with either HA-tagged Wnt1 expression vector (pHA-Wnt1) or empty vector (pcDNA3) *via* hydrodynamic tail vein injection at 2 days before BIRI (Figure 1A). Serum levels of creatinine and BUN, two biomarkers of kidney injury, were significantly increased in mice 1 day after BIRI, suggesting the existence of AKI (Figures 1B,C). Consistently, prominent kidney morphologic lesions, such as tubular injuries in the corticomedullary junction, were observed in AKI mice (Figure 1D). Notably, these alterations in serum kidney injury biomarkers and morphologic lesions in AKI mice were markedly attenuated by exogenous Wnt1. Western blot analyses revealed that exogenous Wnt1 promoted the expression of renal β-catenin in AKI mice (Figures 1E,F). Taken together, these findings suggest that pre-treatment of exogenous Wnt1 activates renal β-catenin and prevents AKI in mice.

### *In vivo* Expression of Exogenous Wnt1 Before IR Reduces Tubular Cell Apoptosis and Inhibits Nuclear Factor-Kappa B Activation

**FIGURE 2:**
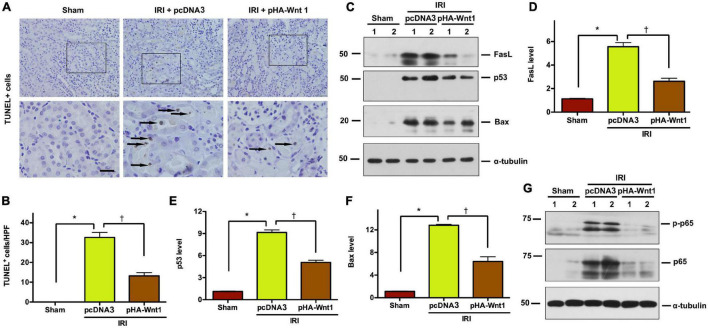
*In vivo* expression of exogenous Wnt1 before IR reduces tubular cell apoptosis and NF-κB activation in AKI mice. **(A)** Representative micrographs show TUNEL-positive cells in different groups as indicated. Arrows indicate positive staining. Scale bar, 100 μm. **(B)** Graphic presentation shows the TUNEL-positive cells per high power field (HPF) in different groups as indicated. **(C–F)** Representative Western blot analyses show renal expression of FasL, p53, and Bax in different groups as indicated. *P < 0.05 versus Sham controls; ^†^*P* < 0.05 versus IRI injected with pcDNA plasmids (*n* = 5–6). **(G)** Representative Western blot of p65 and p-p65 proteins in different groups as indicated.

To explore whether regulation of apoptosis is the mechanism underlying the protective effect of exogenous Wnt1 on AKI, TUNEL staining was performed to identify apoptosis in kidney tissue of AKI mice. Compared with Sham mice, IRI markedly increased the apoptosis rate as well as the levels of apoptosis-related proteins, such as FasL, p53 and Bax, in kidneys of mice (Figures 2A–F). Such alterations in kidneys of AKI mice were markedly reduced by *in vivo* expression of exogenous Wnt1 before IR. Nuclear factor-kappa B (NF-κB), particularly its heterodimer form p65/p50, plays a critical role in regulating the inflammatory response in kidneys of AKI mice. Western blot analysis showed that both p65 and its phosphorylated form (p-p65) were markedly increased in kidneys of mice after IRI, whereas such alterations were prevented by exogenous Wnt1 (Figure 2G). These findings suggest that *in vivo* expression of exogenous Wnt1 before IR prevents apoptosis and inhibits NF-κB activation in kidneys of AKI mice.

### *In vivo* Expression of Exogenous Wnt1 Before IR Prevents the Progression of Acute Kidney Injury to Chronic Kidney Disease

**FIGURE 3:**
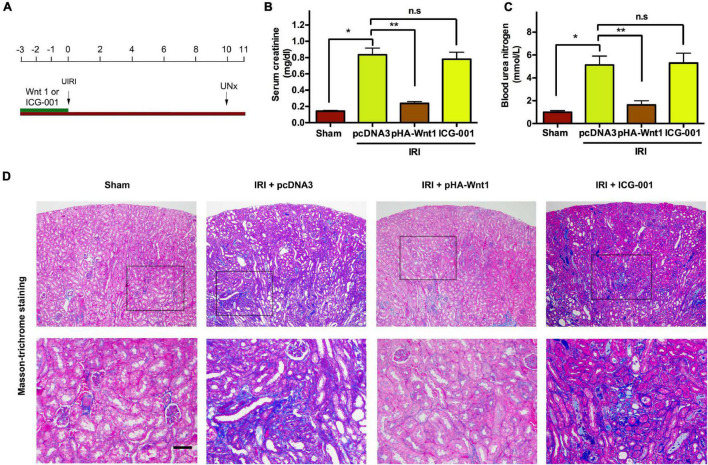
*In vivo* expression of exogenous Wnt1 before IR prevents the progression of AKI to CKD in mice. **(A)** Mouse treatment groups. Mice were subjected to either hydrodynamic tail vein injection of 1 mg/kg pcDNA3 (IRI + pcDNA3) or pHA-Wnt1 (IRI + pHA-Wnt1) plasmid, or intraperitoneal injection of 5 mg/kg ICG-001 at 3 days before UIRI. Blood and tissues were collected at 11 days after UIRI. Serum creatinine levels **(B)** and blood urea nitrogen (BUN) levels **(C)** were measured. **(D)** Representative micrographs of kidney morphology at 11 days after UIRI. Kidney sections were subjected to Periodic acid–Schiff staining. Boxed areas are enlarged. Arrows indicate renal tubules. Scale bar, 100 μm. *P < 0.05; **P < 0.01. *n* = 5.

We next examined whether activation of Wnt/β-catenin before IR can prevent the progression of AKI to CKD. As shown in Figure 3A, mice underwent either hydrodynamic tail vein injection of Wnt1 expression plasmid (pHA-Wnt1) or intraperitoneal injection of ICG-001 at 3 days before UIRI. Both serum creatinine and BUN levels were increased in mice at 11 days after UIRI (Figures 3B,C). Additionally, Masson trichrome staining revealed prominent renal collagen deposition and fibrotic lesions in mice at 11 days after UIRI (Figure 3D). These observations indicate the progression of AKI to CKD in mice after UIRI. Notably, the observed alterations of serum kidney injury biomarkers and renal fibrosis in CKD mice were almost completely prevented by pre-treatment of exogenous Wnt1, but not ICG-001. Therefore, *in vivo* expression of exogenous Wnt1 before IR prevents the progression of AKI to CKD in mice.

### *In vivo* Expression of Exogenous Wnt1 Before IR Down-Regulates Endogenous Wnt1 and β-Catenin in Kidneys

**FIGURE 4:**
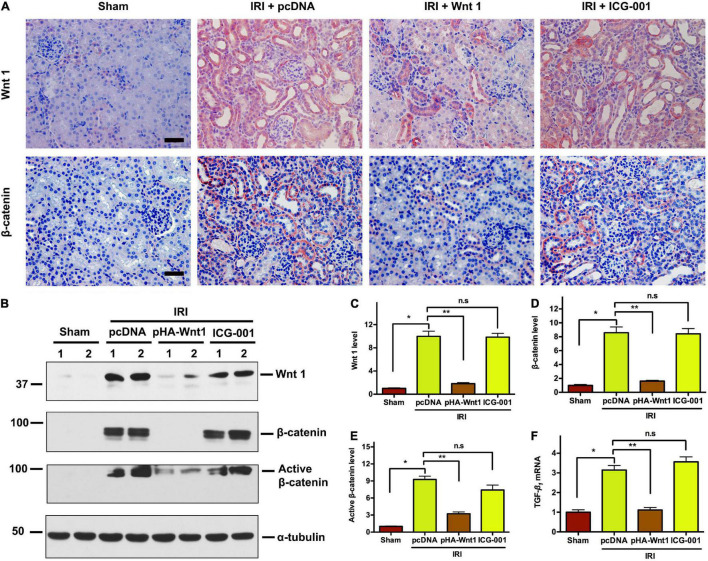
*In vivo* expression of exogenous Wnt1 before IR down-regulates endogenous Wnt1 and β-catenin in kidneys of mice after AKI-CKD progression. **(A)** Representative micrographs show Wnt and β-catenin protein expression in different groups as indicated. Scale bar, 50 μm. **(B–E)** Representative Western blot analyses of Wnt1, β-catenin, and active β-catenin protein levels in different groups as indicated. **(F)** mRNA expression of TGF-β in different groups as indicated. *P < 0.05; **P < 0.01. *n* = 5.

We further investigated the effect of exogenous Wnt1 on endogenous Wnt/β-catenin signaling in kidneys of CKD mice. As shown in Figure 4A, immunohistochemical staining revealed that both endogenous Wnt1 and β-catenin were markedly up-regulated in kidneys of mice at 11 days after IRI. Their protein levels were greatly reduced by pre-treatment of exogenous Wnt1, but not ICG-001. Western blot analyses confirmed the inhibitory effect of exogenous Wnt1, but not ICG-001, on endogenous expression of Wnt1, β-catenin and active β-catenin in kidneys of CKD mice (Figures 4B–E). The Wnt/β-catenin and TGF-β signaling pathways crosstalk in renal fibrosis. The mRNA expression of TGF-β was induced in kidneys of CKD mice, and such induction was diminished by exogenous Wnt1, but not ICG-001 (Figure 4F). Therefore, pre-treatment of exogenous Wnt1 inhibits endogenous renal Wnt/β-catenin signaling in CKD mice.

### *In vivo* Expression of Exogenous Wnt1 Before IR Downregulates Renal Wnt/β-Catenin Target Genes in Mice

**FIGURE 5:**
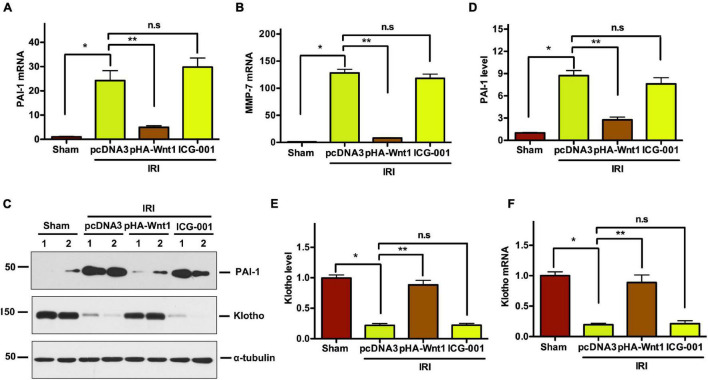
*In vivo* expression of exogenous Wnt1 before IR downregulates renal Wnt/β-catenin target genes in mice after AKI-CKD progression. **(A,B)** mRNA expression of PAI-1 and MMP-7 in different groups as indicated. **(C–E)** Representative Western blot analyses of PAI-1 and Klotho protein levels. **(F)** mRNA expression of Klotho. *P < 0.05; **P < 0.01. *n* = 5.

Compared with Sham mice, the mRNA expression of PAI-1 and MMP-7, two direct downstream targets of Wnt/β-catenin signaling, was significantly up-regulated in kidneys of mice at 11 days after UIRI (Figures 5A,B). Pre-treatment of exogenous Wnt1, but not ICG-001, inhibited PAI-1 and MMP-7 mRNA expression in CKD mice. Western blot analysis showed that PAI-1 protein levels were increased in kidneys of CKD mice, where were abolished by pretreatment of exogenous Wnt1, but not ICG-001 (Figures 5C,D). Klotho is an endogenous Wnt antagonist by binding and sequestering Wnt ligands. Compared with Sham mice, both mRNA and protein levels of Klotho were decreased in kidneys of mice at 11 days after UIR (Figures 5E,F). Pre-treatment of exogenous Wnt1, but not ICG-001, almost completely restored Klotho mRNA and protein expression in kidneys of CKD mice. These findings further suggest the inhibitory effect of exogenous Wnt1 on endogenous Wnt/β-catenin signaling in kidneys of CKD mice.

### *In vivo* Expression of Exogenous Wnt1 Before IR Reduces Renal Fibrosis in Mice After Acute Kidney Injury-Chronic Kidney Disease Progression

**FIGURE 6:**
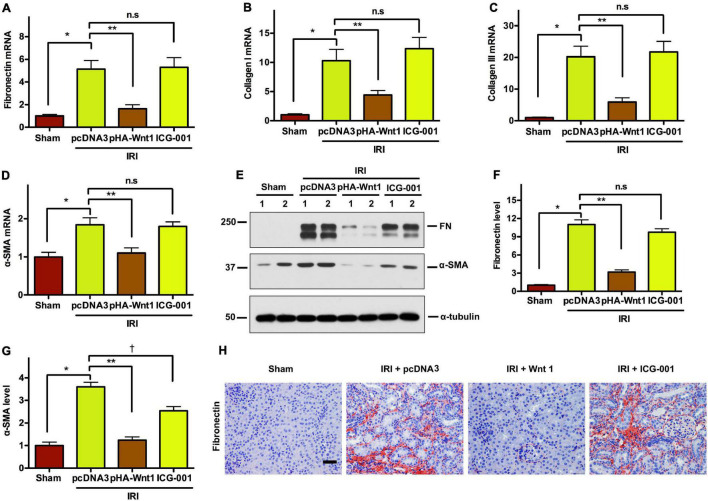
*In vivo* expression of exogenous Wnt1 before IR reduces renal fibrosis in mice after AKI-CKD progression. **(A–D)** mRNA expression of fibronectin, collagen I, collagen III, and α-SMA. **(E–G)** Representative Western blot analyses of fibronectin and α-SMA-SMA protein levels. **(H)** Immunostaining of fibronectin in kidney sections. *P < 0.05; **P < 0.01; ^†^*P* < 0.05. *n* = 5. Scale bar, 50 mm.

We next investigated the effect of Wnt/β-catenin activation before IR on renal matrix genes and fibrotic lesions in CKD mice. Compared with Sham mice, the mRNA expression of fibronectin (FN), collagen I, collagen III, and α-SMA was markedly induced in kidneys of mice at 11 days after UIR (Figures 6A–D). Such alterations were diminished by pre-treatment of exogenous Wnt1, but not ICG-001. Western blot analysis showed that the protein levels of FN and α-SMA were significantly increased in kidneys of CKD mice, and such alterations were abolished by pre-treatment of exogenous Wnt1, but not ICG-001 (Figures 6E–G). The effects of exogenous Wnt1 and ICG-001 on FN protein expression in CKD mice were validated by kidney immunostaining with FN antibody (Figure 6H). These findings suggest that pre-treatment of exogenous Wnt1 prevents renal fibrogenesis in IR-induced CKD mice.

### *In vivo* Expression of Exogenous Wnt1 Before IR Attenuates Renal Inflammation After Acute Kidney Injury-Chronic Kidney Disease Progression

**FIGURE 7:**
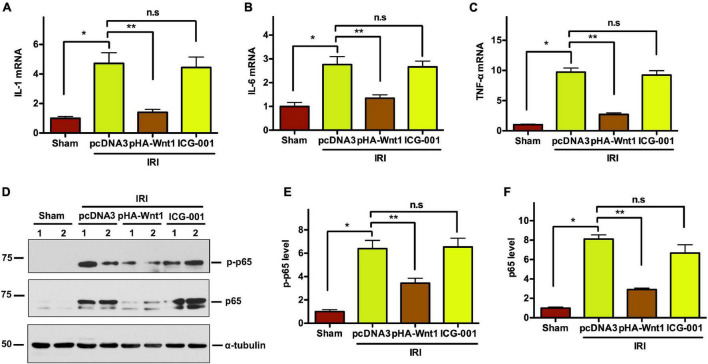
*In vivo* expression of exogenous Wnt1 before IR attenuates renal inflammation in mice after AKI-CKD progression. **(A–C)** mRNA expression of IL-1, IL-6, and TNF-α in kidneys of mice. **(D–F)** Representative Western blot analyses of p65 and p-p65 protein levels. *P < 0.05; **P < 0.01. *n* = 5–6.

Compared with Sham mice, the mRNA expression of inflammatory genes, such as IL-1, IL-6, and TNF-α, was significantly up-regulated in kidneys of mice at 11 days after UIR (Figures 7A–C). Such alterations were almost completely abolished by pre-treatment of exogenous Wnt1, but not ICG-001. Western blot analysis showed that the protein levels of both P65 and its phosphorylated form (p-p65) were markedly increased in kidneys of mice at 11 days after UIRI, indicating the activation of NF-κB signaling in CKD mice (Figures 7D–F). Notably, pre-treatment of exogenous Wnt1, but not ICG-001, significantly reduced p65 and p-p65 protein levels in kidney of CKD mice. These findings indicate that pre-treatment of exogenous Wnt1 prevents renal inflammatory responses in CKD mice.

### Exogenous Wnt1 Protects Tubular Cells Against Apoptosis Induced by Hypoxia-Reoxygenation Injury *in vitro*

**FIGURE 8:**
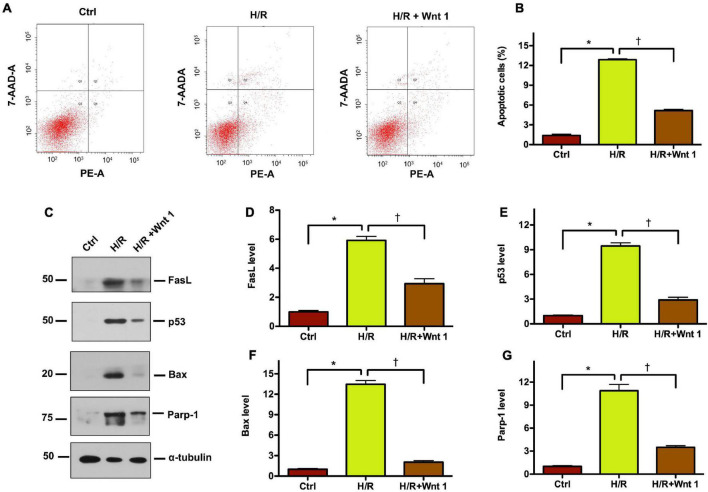
Exogenous Wnt1 protects tubular cells against apoptosis induced by hypoxia-reoxygenation (H/R) injury *in vitro.* **(A)** Representative FACS analyses show that Wnt1 inhibited hypoxia/reoxygenation (H/R)-induced cell apoptosis. HKC-8 cells were pre-expressed with exogenous Wnt1, followed by incubation in hypoxic condition for 48 h and then reoxygenation for 2 h. **(B)** Graphic presentation shows the percentage of apoptotic cells in different groups as indicated. The PE-labeled Annexin V-positive cells were counted by flow cytometry. *P < 0.05 versus controls; ^†^*P* < 0.05 versus H/R (*n* = 3). **(C)** Representative Western blot analyses show protein expression of FasL, p53, Bax, and Parp-1 in HKC-8 cells. **(D–G)** Graphic presentations show the relative abundances of FasL, p53, Bax, and Parp-1 in HKC-8 cells. *P < 0.05 versus controls; ^†^*P* < 0.05 versus H/R (*n* = 3).

To further pinpoint the role of Wnt1 in AKI, we established HKC-8 tubular cell models of AKI using hypoxia-reoxygenation (H/R) treatment. Flow cytometry was employed to detect the apoptosis, and revealed the elevation of apoptosis rate in HCK-8 cells after H/R treatment (Figures 8A,B). Exogenous Wnt1 markedly reduced apoptosis in HCK-8 cells after H/R treatment. Western blot analyses showed that H/R treatment significantly increased the levels of apoptosis-related proteins, such as FasL, p54, Bax, and Parp-1 in HCK-8 cells (Figures 8C–G). Such alterations were diminished by exogenous Wnt1. These data suggest that exogenous Wnt1 protects against H/R-induced apoptosis in tubular cells.

## Discussion

Although AKI is increasing in incidence and is the major risk factor for progression to CKD, there are no effective therapies for AKI prevention and treatment. A number of situations, such as heart surgery, intra-tubular deposits, and administration of nephrotoxic drugs, could lead to the development of AKI (Mas-Font et al., 2017). This highlights the need for preventive measures of AKI.

Although numerous studies support the beneficial effects of Wnt/β-catenin activation in the pathogenesis of AKI, it remains elusive about the preventive effect of Wnt/β-catenin agonists on AKI. One novel finding in the present study is that *in vivo* expression of exogenous Wnt1 before IR could protect mice against AKI and prevent the progression of AKI to CKD. *In vivo* expression of exogenous Wnt1 at 2 days before renal bilateral IR was able to activate renal β-catenin, leading to reduced renal apoptosis and inflammation in AKI mice (1 day after IR). Additionally, *in vivo* expression of exogenous Wnt1 at 3 days before renal unilateral IR inhibited endogenous Wnt/β-catenin signaling and prevented the development of renal fibrosis in CKD mice (11 days after IR). In contrast, *in vivo* expression of exogenous Wnt1 at 5 days after renal IR was shown to induce β-catenin activation and accelerate AKI to CKD progression (Xiao et al., 2016). These studies suggest that the timing of Wnt/β-catenin agonists is an important factor when they are used for AKI prevention and treatment.

Studies suggest that sustained activation of Wnt/β-catenin signaling could drive the progression of AKI to CKD. ICG-001 selectively inhibits Wnt/β-catenin/CREB binding protein (CBP) signaling, and is currently in clinical trial for various cancers. We previously showed that administration of ICG-011 at 5 days after IR restored kidney function and impeded the progression of AKI to CKD, as evidenced by reduced renal fibrosis (Xiao et al., 2016). Additionally, ICG-001 administration starting 3 days after unilateral ureteral obstruction (UUO) was able to attenuate renal fibrotic lesions in UUO models of CKD mice (Hao et al., 2011). However, the present study showed that pre-treatment of ICG-001 at 3 days before IR had no effect on renal Wnt/β-catenin signaling or the progression of AKI to CKD. This is not surprising because Wnt/β-catenin signaling is expressed at very low levels in uninjured adult kidney (Tan et al., 2014). Nonetheless, our findings suggest that the timing and duration of ICG-001 administration are critical to determine its therapeutic outcome.

Mechanisms underlying the protective effect of Wnt/β-catenin may involve the modulation of apoptosis and survival pathways. Genetic ablation of tubule β-catenin promotes tubular apoptosis after IRI or folic acid treatment, whereas activation of Wnt/β-catenin suppresses pro-apoptotic proteins (Wang et al., 2009; Zhou et al., 2012). In the present study, exogenous Wnt1 markedly inhibited tubular cell apoptosis in both IRI mouse models of AKI and H/R-induced cell models of AKI. This suggests that apoptosis could be a potential mechanism underlying the preventive effect of exogenous Wnt1 on AKI and AKI to CKD progression. The present study also demonstrated an inhibitory effect of exogenous Wnt1 on NF-κB activation in both AKI and CKD, indicating a suppression of inflammatory response. However, it should be noted that whether reduced inflammation is a secondary effect to protection or the mechanisms of protection mediated by exogenous Wnt1 remains to be determined. Further studies are needed to elucidate the exact mechanism for exogenous Wnt1-mediated protection against AKI and prevention of AKI to CKD progression.

## Conclusion

In conclusion, the present study provides the evidence to support the preventive effect of Wnt/β-catenin activation on IR-related AKI and CKD, though more work is needed to elucidate the exact mechanisms by which exogenous Wnt1 protects against AKI and CKD. Given the dual role of Wnt/β-catenin signaling in AKI and CKD, the present study highlights the importance of timing when using Wnt/β-catenin modulators for the prevention and treatment of AKI. Future studies using more pharmacologic and genetic approaches as well as the optimal treatment timing and duration are warranted to investigate the effect of Wnt/β-catenin agonists in AKI prevention and treatment.

## Data Availability Statement

The original contributions presented in the study are included in the article/Supplementary Material, further inquiries can be directed to the corresponding author.

## Ethics Statement

The animal study was reviewed and approved by the Animal Ethical and Welfare Committee at Southern Medical University.

## Author Contributions

XH, YZ, and LX conceived and performed the experiments. DW, TG, FL, and YL conducted the experiments. TG, YL, and LX drafted the manuscript. All authors participated in the experiments, and gave final approval to the version submitted for publication.

## Conflict of Interest

The authors declare that the research was conducted in the absence of any commercial or financial relationships that could be construed as a potential conflict of interest.

## Publisher’s Note

All claims expressed in this article are solely those of the authors and do not necessarily represent those of their affiliated organizations, or those of the publisher, the editors and the reviewers. Any product that may be evaluated in this article, or claim that may be made by its manufacturer, is not guaranteed or endorsed by the publisher.
