# Supplementary material for: Exogenous Wnt1 Prevents Acute Kidney Injury and Its Subsequent Progression to Chronic Kidney Disease
Source: Front Physiol. 2021 Nov 8;12:745816. doi: 10.3389/fphys.2021.745816 (PMC8606814; doi:10.3389/fphys.2021.745816)
Supplement: Supplementary file 1 [file Table_1.DOCX]

**Supplemental Table 1 The primer sequences used in the present study.**

| Gene | Primer | Sequence（5’-3’） |
| --- | --- | --- |
| *Collagen I* | Forward | ATCTCCTGGTGCTGATGGAC |
|  | Reverse | ACCTTGTTTGCCAGGTTCAC |
| *Fibronectin* | Forward | CGAGGTGACAGAGACCACAA |
|  | Reverse | CTGGAGTCAAGCCAGACACA |
| *TNF-α* | Forward | ACTCCCAGAAAAGCAAGCAA |
|  | Reverse | CGAGCAGGAATGAGAAGAGG |
| *MMP-7* | Forward | CTGGAGCCCGAAGACCCT |
|  | Reverse | CGTCCACTGTACGTGCAGAAGT |
| *PAI-1* | Forward | TGGTGTAGCCTTCGCAGTCA |
|  | Reverse | CCGTGCATCCGCAAACTC |
| *IL-1* | Forward | CCCTTCCGTCAGGTTGGC |
|  | Reverse | CCTCATCCGTATGTGGCTTG |
| *IL-6* | Forward | ACGCACGAGAAAGGGAACG |
|  | Reverse | GAGGCTACAGGAGCCAGACACT |
| *Actin* | Forward | CAGCTGAGAGGGAAATCGTG |
|  | Reverse | GAGGCTACAGGAGCCAGACACT |
| *Collagen III* | Forward | AGGCAACAGTGGTTCTCCTG |
|  | Reverse | GACCTCGTGCTCCAGTTAGC |
| *α-SMA* | Forward | GAGGCACCACTGAACCCTAA |
|  | Reverse | CATCTCCAGAGTCCAGCACA |
